# Job satisfaction and associated factors among married female nurses at a district hospital in Sri Lanka: a cross-sectional study

**DOI:** 10.1186/s12912-026-04945-w

**Published:** 2026-06-21

**Authors:** Malgoda Gamage Nimesha Madushani Amarasinghe, Samaranayaka Gamage Roshani Erandika, Ahangama Wedage Gilma Sandamali, Welikonthegoda Madusha Kalhari, Dewarahandhi Kavishka Madushan De Silva, Kumarasinghe Arachchigey Sriyani

**Affiliations:** 1 Department of Nursing, Faculty of Health Sciences, The Open University of Sri Lanka, Nawala, Nugegoda, Sri Lanka; 2 Department of Clinical Nursing, Faculty of Nursing, University of Colombo, Nugegoda, Colombo, Sri Lanka

**Keywords:** Job satisfaction, Married female nurses, Associated factors, Sri Lanka

## Abstract

**Background:**

Job satisfaction is a key determinant of nursing performance and retention, yet married female nurses may face unique personal and professional demands that influence their workplace experiences.

**Methods:**

This descriptive cross-sectional study assessed the level of job satisfaction and the personal, work-related, and socio-economic factors associated with job satisfaction among married female nurses working at a District General Hospital in the Southern Province of Sri Lanka. A sample of 306 nurses was selected through simple random sampling, and data were collected using a self-administered questionnaire comprising demographic variables and the validated Nurse Job Satisfaction Scale (ESET). Descriptive statistics and one-way ANOVA were performed using SPSS version 26, with statistical significance set at *p* < 0.05.

**Results:**

A total of 251 nurses responded. Overall job satisfaction was high (132.07 ± 20.64), with the highest satisfaction observed in professional recognition (3.9 ± 0.66) and the lowest in recognition and remuneration (3.00 ± 0.85). Significant associations were identified between job satisfaction and age (*p* = 0.031), workplace unit (*p* = 0.001), and educational level (*p* = 0.010).

**Conclusions:**

These findings underscore the complexity of job satisfaction and highlight the need for further research to explore additional associated factors affecting married female nurses.

**Supplementary Information:**

The online version contains supplementary material available at https://doi.org/10.1186/s12912-026-04945-w.

## Introduction

Nurses constitute the largest segment of the healthcare workforce and play a central role in delivering safe, continuous, and patient-centred care [1, 2]. However, modern nursing is increasingly characterised by significant challenges, including workplace bullying, extended working hours, and the burden of double shifts [3]. These stressors are taking a toll on nurses’ well-being, leading to diminished morale, emotional exhaustion, and decreased job satisfaction [4]. Nurses’ job satisfaction refers to the degree to which nurses like or enjoy the work they do [5]. Job satisfaction plays a crucial role in guiding health care teams as they navigate their work environment. It not only ensures that individuals experience contentment in their roles but also cultivates positive emotions and fosters constructive attitudes [6]. Studies have shown that job dissatisfaction among nurses can lead to increased burnout, absenteeism, turnover, and decreased patient safety [3, 7].

Job satisfaction is a critical factor influencing nurses’ performance, retention, and overall well-being. In India, 75% of nurses reported being satisfied with their jobs, with factors such as sleep, financial needs, and workload significantly influencing their satisfaction [8]. In Sri Lanka, studies on job satisfaction among nurses are limited but reveal important insights. One local study identified workload, professional support, training received, and working conditions as critical factors associated with job satisfaction in government hospitals [9]. Additionally, job satisfaction is found to increase with age and years spent in the current hospital [9]. Despite these findings, dissatisfaction with specific aspects such as wages and overtime shifts remains prevalent. For example, a study at the Teaching Hospitals of Karapitiya and Mahamodara in Sri Lanka found that 67% of nurses worked overtime, and 83% were dissatisfied with their wages [10]. Similarly, another Sri Lankan study found that working conditions, staffing levels, and remuneration significantly affected nurses’ job satisfaction [11].

Globally, research indicates that job satisfaction among nurses is influenced by intrinsic factors such as professional autonomy, recognition, and extrinsic factors such as remuneration, staffing, workload, and organisational support. A study conducted among rural and urban nurses in Ontario, Canada, identified benefits and job security as key contributors to job satisfaction [3]. Similarly, another study reported that job satisfaction plays a significant role in turnover intentions, emphasising the need for healthcare leaders to prioritise satisfaction-enhancing strategies to improve nurse retention [12]. Evidence from the United States further suggests the role of organisational and psychological factors in job satisfaction. Lower levels of autonomy, high psychological demands, and inadequate peer and supervisor support were associated with increased job dissatisfaction [13]. Consistent with these findings, a larger multicountry study across 12 European countries showed that demanding work patterns, particularly long shifts, were associated with lower job satisfaction and increased burnout, emphasising the importance of supportive work environments and manageable workloads [14].

In the Sri Lankan context, a mixed picture emerges from recent studies. One local study reported that only 47% of nurses working in labour and postnatal units expressed satisfaction with socio-demographic factors playing a role. Similarly, findings from another study suggested that recruitment challenges, working conditions, and remuneration remain persistent issues affecting job satisfaction [10]. In many healthcare settings, including in Sri Lanka, the nursing workforce is predominantly female, and most nurses are married [15, 16].

A key limitation in the current literature is the lack of focus on specific subgroups within the nursing workforce, particularly those who may experience unique role-related stressors. One such underexplored group is married female nurses who represent a substantial proportion of the nursing workforce in Sri Lanka [17, 18]. The exclusive focus on nurses in this study is justified by their frontline role in patient care delivery, their continuous exposure to workplace stressors, and their critical influence on the healthcare system and patient safety outcomes. This demographic trend highlights the importance of understanding the specific experiences of married female nurses, given the demands of professional duties and family obligations, which can significantly affect their job satisfaction. Focusing specifically on married female nurses is theoretically and empirically justified, as marital status and gender jointly shape work experiences, role expectations, and stress exposure in the nursing profession. Married female nurses often assume primary responsibility for household management, childcare, and elder care in addition to professional duties, placing them at heightened risk of work–family conflict, emotional exhaustion, and role overload compared to their unmarried counterparts [19]. Empirical evidence indicates that marital quality, spousal support, and family demands are significantly associated with job satisfaction and psychological well-being among married nurses [20].

A previous study in China reported low levels of job satisfaction among married female nurses and a positive relationship between marital quality and job satisfaction [20]. This warrants the importance of understanding job satisfaction among married nurses within the local context. Variations in shift schedules may reduce the time nurses have to spend with their spouses and families, which may, in turn, affect their job satisfaction [20]. Despite this, there is a research gap in the Sri Lankan literature: few studies have specifically examined job satisfaction among married female nurses in this context. Existing studies either generalise findings across all nurses or fail to capture the intersection of gender, marital status, and occupational stressors. This limits the ability to develop targeted, context-sensitive interventions to improve workforce outcomes.

In addition, geographical disparities in research remain evident. Most available Sri Lankan studies are concentrated in teaching or tertiary hospitals. Limited evidence exists from district-level healthcare institutions, which differ in terms of patient load, staffing patterns, and resource availability. General district hospitals also play a vital role in delivering secondary healthcare services and often operate under unique organisational and workforce constraints.

Under this light, the study aims to assess married female nurses’ job satisfaction and associated factors in a district hospital in the Southern District of Sri Lanka. Understanding these dynamics will provide subgroup-specific evidence on job satisfaction, highlighting the influence of gendered and marital roles on work experiences. Secondly, it offers contextual insights from a district-level healthcare setting, which is underrepresented in current research. Third, it adds contextual insight into how personal, professional, and socio-economic factors interact to shape job satisfaction among married female nurses.

## Methods

### Study design and setting

A descriptive cross-sectional study was conducted to assess the job satisfaction of married female nurses at a District General Hospital in the Southern Province of Sri Lanka. This hospital was selected because it is a major government healthcare institution with a large and diverse nursing workforce (*N* = 1000), allowing adequate representation of married female nurses across multiple clinical units. Further, this hospital has high patient turnover, which allowed the inclusion of nurses working in diverse professional environments within the public healthcare system.

### Population

The study population comprised all female, married nurses working at the specified hospital. The inclusion criteria required that participants be female, married, and have at least one year of experience in their current ward or unit. Nurses who were pregnant, on maternity leave, or temporarily assigned to other units were excluded from the study. Pregnant nurses and nurses on maternity leave were excluded because pregnancy and maternity leave are transitional physiological and psychosocial periods that may temporarily affect job satisfaction, independent of workplace factors. Additionally, nurses on maternity leave are not actively exposed to the current work environment, which could affect the accuracy of the job satisfaction assessment. Although the majority of the nursing workforce (*n* = 910; 91.0%) consisted of married female nurses with more than one year of experience, eligibility was confirmed individually after random selection. However, to ensure adequate statistical power, the sample size was calculated based on the total nursing population (*N* = 1000), yielding a conservative estimate.

### Sample size and sampling technique

The sample size was calculated according to the statistical finite population correction formula, $\:n=\frac{{Z}^{2}.P.q.N}{{e}^{2}\:\left(N-1\right)+\:{Z}^{2}.P.q}$

which is widely used to calculate sample sizes in research involving finite populations. Where N = population (1000), “Z = Z-score corresponding to the desired confidence level (95% = 1.96)”, p = sample proportion (0.5), q = 1-p (0.5), and e = acceptable margin of error (0.05). This yielded an estimated sample size of 278 respondents. A 10% (*n* = 27.8 ≈ 28) non-response adjustment was added to account for possible incomplete participation, based on previous survey-based studies among hospital staff, where response rates may vary due to workload and shift duties, resulting in a final sample size of 306 participants. The sample size was calculated to estimate the level of job satisfaction among married female nurses, the study’s primary outcome. Since no previous local data were available on job satisfaction among married female nurses in a district hospital setting, a proportion (*p* = 0.5) was assumed to ensure the maximum sample size and improve precision.

Simple random sampling was performed using the hospital nursing staff registry as the sampling frame. Each nurse was assigned a unique identification number, and participants were selected by lottery. After selection, each selected nurse was assessed against the inclusion criteria. If a selected nurse did not meet the eligibility criteria (for example, being unmarried or having less than one year of work experience), that nurse was excluded and replaced with another nurse randomly selected from the remaining nursing staff list until the required sample size of 306 eligible participants was reached. Because the proportion of ineligible nurses was small, only two nurse replacements were required. Because no baseline demographic information was collected from non-respondents, a formal non-response bias analysis could not be performed.

### Data collection tool

Data were gathered using a self-administered questionnaire (Supplementary file 01) comprising four sections: demographic details, job satisfaction assessment, work-related factors, and socio-economic factors. The independent variables included in this study were selected based on prior empirical evidence, the unique context of married female nurses, and their contextual relevance to the Sri Lankan nursing workforce. Personal variables such as age and number of children were included because they influence work–family balance. Professional variables, including unit of working, years of experience, and position, were selected because of their established association with workload, responsibility, and autonomy. Socio-economic variables, such as educational level, family income, overtime hours, and mode of transport to work, were considered as they may contribute to physical fatigue, commuting burden, time constraints, and work-related stress, which can indirectly influence job satisfaction among shift-working nurses. Personal variables included age and number of children. Age was categorised into four groups (< 30, 31–41, 41–50, and 51–60 years), and the number of children was categorised as none, 1–3, or > 3.

Work-related variables included unit of working (surgical, medical, pediatric, operating theatre, ICU, OPD/ETU, clinics, and other units), nursing position (Grade III, Grade II, Grade I, and Supra Grade), years of nursing experience (< 5, 5–15, 16–25, and > 25 years), and years of experience in the current unit (< 5, 5–10, 11–15, and > 15 years).

Socio-economic variables included highest educational qualification (diploma, undergraduate, graduate, postgraduate), monthly family income (< LKR 125,000; LKR 125,000–175,000; LKR 175,001–250,000; > LKR 250,000), overtime hours per month (< 20, 20–40, 41–60, and 61–80 h), and mode of transport to work (on foot, private vehicle, or public transport). All variables were treated as categorical variables for analysis.

**Fig. 1:**
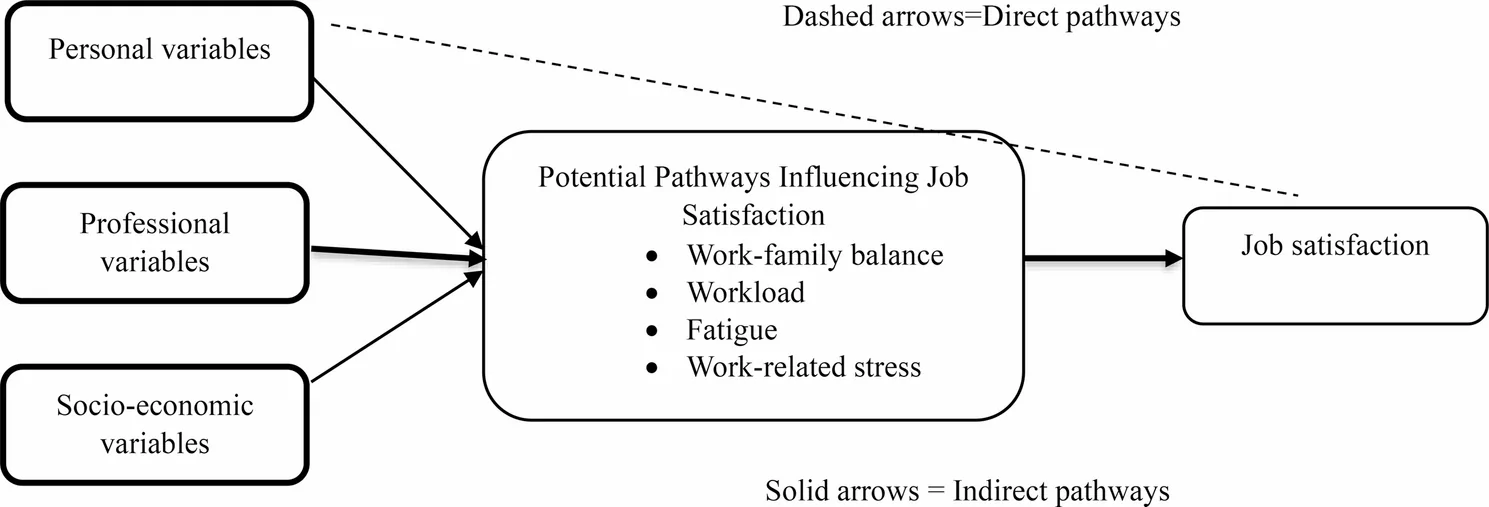
Conceptual framework based on the Job Demands–Resources (JD-R). This model illustrates personal, professional, and socio-economic factors associated with job satisfaction among married female nurses, mediated by work–family balance, workload, fatigue, and work-related stress

The conceptual framework (Fig. 1) illustrates how personal, professional, and socio-economic variables may influence job satisfaction, both directly and indirectly, through work–family balance, workload, fatigue, and work-related stress among married female nurses.

The conceptual framework is primarily grounded in the Job Demands- Resources (JD-R) model [21], which explains how job demands such as workload, fatigue, work-family balance, and work-related stress influence employee outcomes, including job satisfaction. Personal, professional, and socio-economic variables are conceptualised as contextual factors that may shape job demands and available resources. In addition, Role Theory provides further support for the inclusion of work-family balance as a mediating mechanism, particularly among married female nurses who manage multiple roles [22]. Accordingly, the framework illustrates both direct and indirect pathways through which these variables influence job satisfaction among married female nurses.

Nurse Job Satisfaction Scale (Escala de Satisfação dos Enfermeiros com o Trabalho – ESET) [23] was utilised to assess job satisfaction among nurses. The Nurse Job Satisfaction Scale, developed by Joao [24], has been validated and shown to be reliable. This 37-item scale evaluates nurses’ satisfaction across various aspects of work dynamics. The survey comprises six dimensions: satisfaction with the co-workers (5 items), satisfaction with recognition and remuneration (5 items), satisfaction with leadership (12 items), satisfaction with staffing (2 items), satisfaction with organisation and resources (8 items), and satisfaction with professional recognition (5 items) [22].

Responses on the scale are rated using a five-point Likert scale, where [1] represents “not at all,” [2] “slightly,” [3] “moderately,” [4] “very,” and [5] “extremely.” A weighted mean scoring system was used to interpret the results, with corresponding score ranges for satisfaction levels. Average scores of 1.00 to less than 1.80 (mean score range: 37 to < 66.6) indicate a very low level of satisfaction, while scores from 1.80 to less than 2.60 (mean score range: 66.6 to < 96.2) reflect a low level of satisfaction. Scores between 2.60 and less than 3.40 (mean score range: 96.2 to < 125.8) indicate a moderate level of satisfaction, and scores of 3.40 to less than 4.20 (mean score range: 125.8 to < 155.4) reflect a high level [22]. Finally, scores from 4.20 to 5.00 (mean score range: 155.4 to 185) represent a very high level of satisfaction. The scale demonstrates excellent reliability, with a Cronbach’s alpha coefficient of 0.92.

Although ESET has not been formally validated in the Sri Lankan context, it was selected for its comprehensive theoretical foundation, multidimensional structure, and strong psychometric performance in prior research. The instrument was administered in English, which is the standard language of nursing education, training, and professional communication in Sri Lanka. Nursing curricula, clinical documentation, and licensing examinations are predominantly conducted in English, suggesting a baseline level of English proficiency among participants. Therefore, linguistic adaptation was not performed.

To ensure contextual appropriateness, the questionnaire underwent content validation by three experts in nursing education and research methodology, who evaluated clarity, cultural relevance, and suitability for the Sri Lankan nursing context. Minor wording adjustments were made for clarity without altering the original structure or meaning of the items.

A pre-test was conducted with 10% (*n* = 30) of the calculated sample size among nurses working in the same hospital setting. Participants in the pre-test were excluded from the main study to prevent contamination. The average time required to complete the questionnaire was approximately 15–20 min. Internal consistency reliability was assessed during the pre-test using Cronbach’s alpha, which indicated acceptable reliability (α > 0.70) across the scale, confirming the instrument’s suitability for the main study.

In the present study, internal consistency reliability was assessed using Cronbach’s alpha coefficient for the overall scale and its subscales to confirm reliability within the study sample. For the overall scale, Cronbach’s alpha was 0.890. The subscale “satisfaction with leadership” demonstrated high internal consistency (α = 0.831), followed by “satisfaction with the organisation and resources” (α = 0.806). The remaining subscales showed acceptable reliability, including satisfaction with professional recognition (α = 0.706), satisfaction with co-workers (α = 0.700), satisfaction with recognition and remuneration (α = 0.710), and satisfaction with staffing (α = 0.700).

## Data collection

After obtaining ethical clearance from the Ethics Review Committee of the Faculty of Medicine, University of Ruhuna, Sri Lanka, data collection was conducted from the 30th of July to the 31st of August 2024. All participants were informed of the research purpose before the questionnaire was distributed to potential participants. The study information sheet was provided to the participants, who were given ample time to read it. Written informed consent was then obtained from those who wished to participate in the study. Data collection was conducted using self-administered questionnaires distributed in sealed envelopes during duty hours at times that did not interfere with patient care, with prior permission from the nursing administration. Participants were allowed to complete the questionnaire at their convenience, either during designated break periods or after duty hours. Completed questionnaires were returned in sealed envelopes to a designated collection box placed in the nursing office of each unit. During the data collection process, participants’ anonymity and confidentiality were protected by assigning unique code numbers rather than using their personal names or other identifying information. Voluntary participation was encouraged. Data security was ensured by limiting access to the collected data to the researchers. Computerised data was password-protected, and physical data forms were kept under lock and key. After five years, electronic data will be permanently deleted, and physical data will be shredded and destroyed.

### Data analysis

All returned questionnaires were reviewed for completeness prior to analysis. Questionnaires with incomplete responses were excluded from the final analysis. Therefore, no missing data imputation procedures were required. Data analysis was conducted using the Statistical Package for the Social Sciences (SPSS), version 26. Descriptive statistics, including percentages, frequencies, means, weighted means, and standard deviations were employed to summarise sociodemographic characteristics and the levels of study variables. The relationships between study variables were examined using one-way ANOVA. When ANOVA results were statistically significant, Bonferroni-adjusted post hoc analyses were conducted to identify which specific group comparisons differed significantly while controlling for multiple comparisons. Before conducting one-way ANOVA, the assumptions of normality and homogeneity of variances were assessed. Normality of the dependent variable within groups was evaluated using the Shapiro–Wilk test and visual inspection of Q–Q plots. Homogeneity of variances was assessed using Levene’s test. Since the assumptions were satisfactorily met, one-way ANOVA was considered appropriate for the analysis. A significance level of *p* < 0.05 was used.

## Results

Of the 306 nurses invited to participate, 251 participated in the study, resulting in a response rate of 82.03%. No demographic or baseline data were available for non-respondents, so formal non-response analysis was not possible. Table 1 presents the socio-demographic profile of the participants. Of the participants, more than half (53%) were aged between 31 and 41 years old. The majority of participants had a Diploma in Nursing (73.3%), with 14.7% having graduated. Grade I nurses were the largest group in the sample (44.1%), while more than half of the participants (59.4%) reported having 5–15 years of experience as nursing officers. However, most participants (49%) had fewer than 5 years of experience in their current work unit. The majority of participants (66.9%) used public transport to report to duty.

**Table 1:** Socio-demographic characteristics of the participants (*N* = 251)

Characteristics	Category	Frequency (*n*)	Percentage (%)
Age (years)	<30	10	4.0
	31–41	133	53.0
	41–50	83	33.1
	51–60	25	10.0
Unit of work	Surgical	38	15.1
	Medical	40	15.9
	Pediatric	24	9.6
	Operation theatre	22	8.8
	ICU	35	13.9
	OPD ETU	10	4.0
	Clinics	25	10.0
	Other	57	22.7
Highest level of education	Diploma	184	73.3
	Undergraduate	29	11.6
	Graduated	37	14.7
	Postgraduate	01	0.4
Position	Grade 3 RN	26	10.4
	Grade 2 RN	84	33.5
	Grade 1 RN	111	44.1
	Supra grade	30	12.0
Experience as a nurse (years)	< 5	06	2.4
	5–15	149	59.4
	16–25	76	30.3
	> 25	20	8.0
Experience as a nurse in the current unit (years)	< 5	123	49.0
	5–10	104	41.4
	11–15	16	6.4
	> 15	08	3.2
Number of children	< 1	22	8.8
	1–3	227	90.4
	> 3	02	0.8
Mode of transport to work	On foot	16	6.4
	Private vehicle	67	26.7
	Public transport	168	66.9

ICU; intensive care unit OPD ETU; outpatient department emergency treatment unit RN; registered nurse

### Job satisfaction among married female nurses

As shown in Table 2. Nurses’ overall job satisfaction level was high with a 132.07 (± 20.64) mean score and 3.56 (± 0.55) weighted mean value. Almost all dimensions of job satisfaction, except for “Satisfaction with recognition and remuneration” and “Satisfaction with staffing,” were reported as at a high level. The highest job satisfaction weighted mean was reported in “Satisfaction with professional recognition” (3.9 ± 0.66), while the lowest weighted mean was reported for “Satisfaction with recognition and remuneration” (3.00 ± 0.85).

**Table 2:** Job satisfaction of participants

Job satisfaction sub-scales	Mean (SD)	Weighted mean (SD)	Level
Satisfaction with the co-workers	19.07 (± 4.01)	3.81 (± 0.80)	High
Satisfaction with recognition and remuneration	15.01 (± 4.28)	3.00 (± 0.85)	Medium
Satisfaction with leadership	43.55 (± 7.70)	3.62(± 0.64)	High
Satisfaction with staffing	6.66 (± 2.19)	3.33 (± 1.09)	Medium
Satisfaction with the organisation and resources	27.94 (± 5.68)	3.49 (± 0.71)	High
Satisfaction with professional recognition	19.81 (± 3.33)	3.9 (± 0.66)	High
**Overall job satisfaction**	**132.07 (± 20.64)**	**3.56 (± 0.55)**	**High**

Table 3 illustrates the levels of overall job satisfaction among nurses. The majority of nurses (46.6%) reported a high level of job satisfaction, with mean scores ranging from 125.8 to less than 155.4. A medium level of satisfaction, with mean scores between 96.2 and less than 125.8, was reported by 33.5% of participants. Additionally, 14.3% of nurses indicated a very high level of satisfaction, with scores ranging from 155.4 to 185. A low level of satisfaction (66.6 to < 96.2) was reported by 5.6% of nurses, while none of the participants fell into the very low satisfaction category (37 to < 66.6).

Although satisfaction with recognition and remuneration was rated at a medium level (weighted mean = 3.00), higher satisfaction scores on other sub-scales, particularly co-workers, leadership, professional recognition, and organisational resources, contributed to a high overall job satisfaction rating (weighted mean = 3.56).

**Table 3:** Levels of overall job satisfaction among nurses

Overall job satisfaction level	Mean range	Frequency (*n*)	Percentage (%)
Very low	37 to less than 66.6	00	00
Low	66.6 to less than 96.2	14	5.6
Medium	96.2 to less than 125.8	84	33.5
High	125.8 to less than 155.4	117	46.6
Very high	155.4 to 185	36	14.3

### Factors associated with nurses’ overall job satisfaction

Table 4 outlines the factors associated with nurses’ overall job satisfaction. Age showed a statistically significant difference in mean job satisfaction scores, *F*(3,247) = 2.995, *p* = 0.031, with a moderate effect size (η² = 0.113). Bonferroni-adjusted post hoc analysis demonstrated that nurses aged 51–60 years had significantly higher satisfaction scores than those aged 41–50 years (mean difference = 12.63, *p* = 0.043). The unit of work also showed a significant group difference, *F*(7,243) = 3.486, *p* = 0.001, with a moderate effect size (η² = 0.091). Post hoc tests showed that nurses working in the ICU had significantly higher total scores than those in the medical ward (mean difference = 16.57, *p* = 0.011) and the pediatric ward (mean difference = 21.63, *p* = 0.002). Educational level was another associated factor (*p* = 0.010), although the observed effect size was small to moderate (η² = 0.045), indicating a comparatively weak practical effect: nurses with diplomas reported higher satisfaction (134.08 ± 20.22) than undergraduates (120.34 ± 18.24).

**Table 4:** Factors associated with nurses’ overall job satisfaction

Characteristics	Category	Mean (SD)	F	η²	*p*
Age (years)	20–30	134.90 (± 13.21)	2.995	0.113	**0.031**
	31–41	133.36 (± 20.93)			
	41–50	127.28 (± 19.77)			
	51–60	139.92 (± 20.64)			
Unit of work	Surgical	132.84 (± 19.82)	3.486	0.091	**0.001**
	Medical	126.77 (± 19.22)			
	Pediatric	121.70 (± 17.99)			
	Operation theatre	129.31 (± 19.73)			
	ICU	143.34 (± 17.62)			
	OPE ETU	134.46 (± 19.27)			
	Clinics	138.88 (± 24.52)			
	Other	132.07 (± 20.64)			
Highest level of education	Diploma	134.08 (± 20.22)	3.850	0.045	**0.010**
	Undergraduate	120.34 (± 18.24)			
	Graduated	131.29 (± 22.15)			
Position	Grade 3 RN	128.15 (± 16.75)	0.574	0.007	0.633
	Grade 2 RN	133.94 (± 20.03)			
	Grade 1 RN	131.46 (± 21.91)			
	Supra grade	132.46 (± 20.94)			
Experience as a nurse (years)	< 5	135.83 (± 8.01)	0.930	0.012	0.383
	5–15	132.96 (± 20.29)			
	16–25	128.92 (± 21.66)			
	> 25	136.25 (± 21.55)			
Mode of transport to work	On foot	123.87 (± 24.00)	1.873	0.015	0.156
	Private vehicle	134.79 (± 20.89)			
	Public transport	132.76 (± 20.11)			
Family monthly income (LKR)	< 125,000	135.02 (± 22.63)	0.551	0.007	0.648
	125,000–175,000	132.25 (± 20.07)			
	175,001–250,000	131.79 (± 21.00)			
	> 250,000	128.00 (± 17.00)			
Overtime hours (per month)	> 20	136.33 (± 22.23)	1.995	0.024	0.115
	20–40	133.20 (± 21.76)			
	41–60	127.18 (± 20.98)			
	61–80	134.07 (± 20.64)			

ICU; intensive care unit OPD ETU; outpatient department emergency treatment unit RN; registered nurse. Values are presented as mean ± standard deviation. One-way ANOVA was used. Bonferroni post-hoc test applied. Means sharing the same superscript letter are not significantly different at *p* < 0.05

## Discussion

Job satisfaction is a critical determinant of workforce retention in nursing and in any other health professions. It influences both employee well-being and quality of patient care. To the best of the authors’ knowledge, few studies have specifically focused on this population in the Sri Lankan context. The present findings highlight three key patterns. First, overall job satisfaction was high despite the moderate dissatisfaction with remuneration and staffing. Second, interpersonal and organisational factors, such as leadership, co-worker relationships, and professional recognition, contributed positively to job satisfaction. Third, selected personal and professional characteristics, including age, working unit, and educational level, were significantly associated with job satisfaction. These findings collectively suggest that job satisfaction among nurses is shaped by a complex interaction between individual and workplace factors rather than by a single association.

The observed high levels of job satisfaction, despite dissatisfaction with remuneration and staffing, can be interpreted through the JD-R Model [21], which postulates that job resources can buffer the negative effects of job demands. In this context, although staffing shortages and inadequate remuneration represent significant job demands, the presence of strong interpersonal and organisational resources, including supportive leadership, positive co-worker relationships, and recognition, may have compensated for these limitations, thereby sustaining overall job satisfaction. This finding is consistent with previous studies conducted in Sri Lanka [9], suggesting that contextual factors, including workplace culture and collegial support, may play a particularly important role in shaping job satisfaction in resource-constrained healthcare settings.

Focusing on the present study on married female nurses is particularly relevant, as they often navigate dual responsibilities between professional duties and family roles, which can influence job satisfaction and overall well-being [19, 23]. This dynamic can be further understood through Role Theory [25], which explains how competing role expectations may lead to role conflict and increased stress. However, the findings of the present study indicate that such potential conflicts do not necessarily translate into lower job satisfaction, likely due to supportive workplace environments. Previous evidence [7] demonstrates that effective leadership and supervisor support play a crucial role in helping nurses to manage work-family demands, while positive co-worker relationships provide emotional and practical support that can buffer work-related stress. These mechanisms may reduce the strain associated with multiple role expectations, enabling married nurses to maintain favourable perceptions of their work.

The present findings demonstrated generally favourable levels of job satisfaction among nurses, with more than half of participants reporting high or very high satisfaction. These findings suggest that nurses in the study setting generally perceived their work environment and professional roles positively. Nevertheless, approximately one-third of respondents reported moderate levels of satisfaction, indicating that important areas requiring organisational attention still exist. Similar findings have been reported in Sri Lanka, where nurses working in the tertiary healthcare settings were predominantly categorised as “satisfied” or “more satisfied” with their jobs [11]. In contrast, studies conducted in other contexts have demonstrated comparatively lower levels of job satisfaction. Such differences may reflect variations in healthcare systems, organisational culture, workforce expectations, staffing patterns, remuneration systems, and professional autonomy across healthcare settings [11, 26].

High levels of satisfaction were observed regarding co-worker relationships, leadership, professional recognition, organisational support, and resource availability, suggesting that the interpersonal and organisational aspects of the work environment were generally perceived positively by nurses in the present study. Previous literature consistently demonstrates that nurses’ job satisfaction is influenced by workplace relationships, leadership practices, organisational culture, and individual factors [11, 26]. Recent evidence further suggests that organisational and leadership factors still remain among the main causes of nurses’ workplace satisfaction, engagement, and retention intentions [27]. Positive relationships with co-workers may contribute to stronger teamwork, emotional support, communication, and resilience in demanding clinical environments. Supportive collegial relationships have been linked to reduced workplace stress, improved work experiences, and greater intention to remain within organisations, particularly in resource-limited settings where teamwork is essential for maintaining quality patient care [26]. Similarly, the high level of satisfaction observed with leadership in the present study may reflect the importance of supportive supervision, communication, and leadership practices in shaping positive workplace experiences. Previous evidence indicates that positive nursing leadership styles are related to improved work engagement, workplace well-being, organisational commitment, and job satisfaction among nurses [27, 28]. Professional recognition and organisational support may also strengthen nurses’ perception of value, professional identity, and commitment toward their organisations. Meaningful recognition and supportive organisational cultures have been connected with increased workforce engagement and more favourable work experiences among nursing staff [29]. Additionally, sufficient organisational resources may reduce work-related frustrations and facilitate the delivery of safe and efficient care, thereby improving workplace perceptions. Studies have revealed that a healthy work environment is characterised by supportive management, effective communication, teamwork, and greater workplace satisfaction [30].

Importantly, these findings provide contextual understanding beyond reporting overall satisfaction levels within a different geographical setting. The observed pattern suggests that nurses’ workplace experiences are shaped by interactions among organisational structures, interpersonal relationships, and the professional environment rather than by single workplace factors alone. The multidimensional pattern may be particularly relevant to resource-constrained healthcare settings, where positive leadership, teamwork, and organisational support may partially offset challenges affected by workload pressures, poor staffing, or financial restrictions. Therefore, present findings highlight the importance of strengthening workplace culture, leadership capacity, professional recognition mechanisms, and organisational support systems when developing policies and strategies for workplace well-being.

Furthermore, present findings are consistent with contemporary theoretical and empirical evidence emphasising the importance of recognition, leadership, and supportive work environments in enhancing employee engagement and satisfaction. Regarding professional recognition, the findings of the present study align closely with the work of Jo and Shin [31], who reported a substantial positive effect of recognition on employee engagement. The findings strongly support Herzberg’s Two-Factor Theory [32], which identifies recognition as a key motivator that enhances job satisfaction and productivity. Recognition contributes to employees’ intrinsic motivation by fulfilling psychological needs for appreciation and validation. In the context of nursing, feelings of being valued and acknowledged professional contribution may strengthen commitment, motivation, and overall satisfaction, as reflected in the high satisfaction scores for professional recognition observed in the present study.

The high level of satisfaction reported regarding co-workers is also supported by prior literature. Adriyanto [33] highlighted that co-workers play a crucial role in shaping job satisfaction through daily interactions, collaboration, and mutual support within the organisation. Positive co-worker relationships facilitate teamwork, acceptance, and shared responsibility, which contribute to a supportive work environment. Conversely, negative interpersonal dynamics can undermine satisfaction and morale. The present findings suggest that nurses who experience supportive and collaborative relationships with colleagues are more likely to report higher job satisfaction.

Furthermore, satisfaction with leadership, as observed in the present study, appears to be closely linked with both recognition and co-worker relationships. Yanto & Yandi [34] emphasised that leadership attitudes significantly influence how co-workers interact with one another. Leaders who promote teamwork, fairness, and open communication foster a positive organisational climate [35], which in turn enhances job satisfaction. The high satisfaction scores related to leadership in the present study suggest that effective leadership practices may have contributed to positive interpersonal relationships and a supportive work culture.

In the present study, nurses reported a high level of satisfaction with the organisation and available resources, indicating perceived adequacy of organisational support. This finding is consistent with international studies showing that supportive organisational environment and resource availability are associated with higher nurse job satisfaction [36]. However, contrasting evidence from other settings has reported low satisfaction with organisational resources due to staffing shortages and workload pressures, highlighting the context-dependent nature of this dimension of job satisfaction [14].

In contrast to the present findings, a recent study among Saudi nurses reported inconsistent results across all dimensions compared to the present study, with “Satisfaction with staffing” rated low and the remaining dimensions rated medium [37]. Higher scores on job satisfaction dimensions in the current study reflect more favourable working conditions and better managerial and supportive measures for married female nurses in the hospital. Consistent with the present findings, previous studies have identified a positive relationship between leadership and supervisor support and job satisfaction [7, 38].

Job satisfaction depends on many factors. As revealed in the present study, nurses’ job satisfaction was associated with their age, unit of work, and level of education. The highest level of job satisfaction was reported in the age group of 51–60 years, while the lowest was found among those aged 41–50 years. The moderate effect size observed for age suggests that age-related differences may have meaningful implications for workforce management. Mid-career nurses may experience greater occupational demands and role strain, highlighting the need for targeted supportive interventions, mentorship opportunities, and workload management strategies to improve retention and job satisfaction among this group. As reported by Sridharan et al. [9], nurses with 13–16 years of service in hospitals had the highest workload compared with those with more than 16 years of service. As noted by these authors, senior nurses have greater authority and responsibilities in the workplace, which may be linked to job satisfaction.

As found in the present study, nurses’ unit of work is an important factor in determining their job satisfaction. The highest job satisfaction was reported among ICU nurses, and the lowest among pediatric unit nurses. This variation may be understood through Person-Environment Fit Theory, which proposes that work-related outcomes are influenced by the degree of alignment between employee characteristics, workplace demands, professional expectations, and organisational environment [39]. According to this perspective, nurses working in environments where workplace characteristics align more closely with professional roles, skills, and available resources may report more favourable workplace experiences, including higher job satisfaction.

The explanation for these differences is likely multifactorial rather than attributable solely to staffing patterns. Intensive care settings are often characterised by structured teamwork, closer interdisciplinary collaboration, specialised clinical responsibilities, and greater professional autonomy, all of which may influence nurses’ workplace experiences [3, 37]. Previous literature suggests that supportive practice environments, teamwork, professional autonomy, and collaborative decision-making contribute positively to nurse satisfaction [29]. Additionally, ICU settings generally operate with comparatively lower nurse-to-patient ratios than many general inpatient settings, which may reduce workload burden and support closer patient monitoring [40]. Collectively, these organisational and professional characteristics may create work environments that align more closely with nurses’ professional expectations and competencies, thereby contributing to higher job satisfaction. The present study revealed that nurses’ level of education was significantly associated with their job satisfaction. As revealed, undergraduate nurses reported lower satisfaction than those with a nursing diploma or degree. This contrasts with findings from previous studies reporting lower job satisfaction among nurses without a Bachelor of Nursing degree or a higher nursing degree than among those with lower qualifications [41, 42]. Lower job satisfaction reported among undergraduate nurses in the present study may be related to the problems they face in balancing their work-life and educational commitments. Although the effect size for educational level was comparatively smaller, these findings suggest that nurses pursuing higher education may require additional institutional support to balance academic and professional responsibilities. Flexible scheduling, educational leave policies, and professional development support may help improve satisfaction among academically advancing nurses.

The present study did not reveal any associations between nurses’ grades, the duration of nursing experience, mode of transport to work, or family monthly income or monthly overtime hours. However, some previous studies have shown a connection between the length of nursing experience and nurses’ job satisfaction [43]. Although there is a numerical difference, the lack of statistical significance suggests that mode of transport is not a key factor in job satisfaction among nurses in this sample. As found in the present study, satisfaction scores are quite similar across income groups. It shows that family income does not play a substantial role in determining nurses’ job satisfaction.

Findings of the present study should be interpreted within the broader context of the Sri Lankan healthcare system, which is largely public-sector driven and provides free healthcare to the population despite persistent resource and workforce constraints. Nurses form the backbone of this system and often work in environments characterised by high patient volumes, staffing shortages, and limited financial incentives [44]. In this context, the moderate satisfaction observed with recognition and remuneration in the present study may reflect systemic and policy-level limitations rather than institution-specific deficiencies. These findings suggest the importance of strengthening leadership practices, peer collaboration, professional recognition, and staffing support to improve nurse retention and well-being.

This study contributes contextual, multidimensional evidence on patterns of job satisfaction across workplace domains and clinical settings. This contribution extends beyond describing job satisfaction within a different geographical context. By examining overall job satisfaction alongside multiple subdomains, the study demonstrates that nurses’ workplace experiences are multidimensional and shaped by the interaction of organisational, interpersonal, and professional factors. The findings further identify unit-specific variations and domain-specific differences, suggesting that workplace experiences are not uniform across nursing settings. These contextual insights may support the development of appropriate interventions targeting leadership, staffing practices, workplace environments, professional recognition, and organisational support, rather than relying solely on generalised approaches to improving nurses’ job satisfaction.

In addition, the findings of this study have several implications for hospital administrators and policymakers. Strengthening supportive leadership practices, improving peer collaboration, and establishing structured professional recognition systems may help sustain nurse satisfaction. Moderate dissatisfaction with remuneration and staffing indicates the need for improved workforce planning, fair workload distribution, and review of non-financial incentive mechanisms. Targeted interventions focusing on lower-satisfaction clinical units may further improve retention and quality of nursing care.

Furthermore, the findings hold important implications for nurse retention, work–life balance, and staff well-being. Given that married female nurses frequently balance job responsibilities with family obligations, hospital administrators should consider introducing adjustable scheduling practices, supportive leave policies, and staff wellness programmes to reduce work–family conflict and occupational stress. The moderate dissatisfaction reported regarding staffing and remuneration suggests the need for workforce policies that minimise excessive workload and encourage equitable staff dispersion across clinical units. Policymakers may also use the results to strengthen retention strategies through professional development opportunities, recognition programmes, and workplace environments that foster nurses’ psychological well-being and long-term commitment to the profession. Those initiatives may help to create a more stable nursing workforce and improve the quality of patient care.

### Limitations

As the study included only married nurses, comparisons by marital status were not possible; however, the findings provide important insights into job satisfaction within this understudied group. The data were collected through a self-administered questionnaire; there may be reporting bias. Since the study was conducted in a single government district hospital, the findings may not be directly comparable to nurses working in private healthcare institutions. Future multicenter studies including both public and private hospitals would provide a broader understanding of job satisfaction across healthcare sectors. Although the response rate of this study was satisfactory (82.03%), a proportion of eligible participants did not respond. Because no demographic or baseline information was collected from non-respondents, it was not possible to conduct a formal non-response analysis. Therefore, the presence of non-response bias cannot be completely excluded. Additionally, dependence on self-reported measures may cause response bias due to social desirability, which influences the accuracy of the reported level of job satisfaction. Although this study focused exclusively on married nurses, the limited availability of comparable studies restricted direct comparisons; however, this also emphasises the need for further research examining job satisfaction among married women in the healthcare workforce. Although psychosocial and organisational factors such as work–family conflict, workplace stress, burnout, and institutional support are recognised as important associated factors of job satisfaction among married female nurses, these variables were beyond the scope of the present study. The exclusion of these factors may have reduced the explanatory power of the findings and limited the development of a more comprehensive understanding of job satisfaction. In addition, the present analysis was limited to bivariate statistical methods. Although one-way ANOVA was appropriate for identifying group differences, consistent with the study’s exploratory objectives, the absence of multivariable analysis limited the ability to control for potential confounding variables and to identify independent determinants of job satisfaction. Therefore, the findings should be interpreted as associative rather than predictive.

## Conclusions

This study should be interpreted as an exploratory investigation that provides locally relevant insights into job satisfaction among married female nurses, rather than as evidence of causal relationships. This study examined job satisfaction and its associated factors among married female nurses and found that overall satisfaction was high. This high level of satisfaction was driven primarily by positive interpersonal and organizational factors, including co-worker relationships, leadership, professional recognition, and organizational resources. In contrast, recognition and remuneration, as well as staffing, were rated as moderate level, indicating persistent challenges related to extrinsic and system-level conditions.

However, the age, unit of work, and educational level were significantly associated with job satisfaction in bivariate analyses.

From a practical perspective, the findings highlight the importance of strengthening supportive leadership practices, improving staffing distribution, promoting collaborative workplace cultures, and enhancing professional support mechanisms for nurses. Particular attention may be needed for nurses working in lower-satisfaction clinical units and for nurses balancing professional and educational responsibilities.

At the policy level, district-level healthcare institutions may benefit from workforce strategies that prioritise staff well-being, equitable workload allocation, continuing professional development opportunities, and non-financial recognition systems. Strengthening organisational support within resource-constrained district hospitals may improve nurse retention, workforce stability, and the quality of patient care.

Future multicenter studies involving district and tertiary healthcare institutions are recommended to better understand contextual differences in nurse job satisfaction across Sri Lanka’s healthcare settings. Further research incorporating psychosocial, organisational, and work–family-related variables, using multivariable analytical approaches, would provide a more comprehensive understanding of the factors associated with job satisfaction among married female nurses.

## Supplementary Information

Below is the link to the electronic supplementary material.

Supplementary Material 1

## Data Availability

The datasets generated and/or analyzed during the current study are not publicly available to protect the participants, but are available from the corresponding author on reasonable request.

## Abbreviations

RN: Registered Nurse
ICU: Intensive Care Unit
OPD: Outpatient Department
ETU: Emergency Treatment Unit
ESET: Escala de Satisfação dos Enfermeiros com o Trabalho (Nurse Job Satisfaction Scale)
SD: Standard Deviation
SPSS: Statistical Package for the Social Sciences
