# Supplementary material for: Job satisfaction and associated factors among married female nurses at a district hospital in Sri Lanka: a cross-sectional study
Source: BMC Nurs. 2026 Jun 21;25:797. doi: 10.1186/s12912-026-04945-w (PMC13540823; doi:10.1186/s12912-026-04945-w)
Supplement: Supplementary file 1 [file 12912_2026_4945_MOESM1_ESM.pdf]

Supplementary file 01

QUESTIONNAIRE

Factors affecting job satisfaction among female married nurses at a District General Hospital in Sri Lanka

Section A: Personal Factors

Instructions: Please answer all the questions. Circle the most appropriate answer when relevant.

1. Age in years								
2. Ward / Unit<br>And year of experience in this ward (years)	Surgical Ward	Medical Ward	Pediatric Ward	Operation Theatre	ICU	OPD ETU	Clinic	Others
3. Duration of experience in years								
4. Duration of working in the current unit								
5. Highest education achievement	Diploma	Undergraduate	BSc	MSc				
6. Present position (Grade)	Grade 3	Grade 2	Grade 1	Super Grade				
7. Marital status	Married	Separated	Divorced	Widowed				
8. Type of residence	Hospital quarters	Rented house	Own house					
9. Number of children living with you								
10. Mode of transport to hospital	On foot	Private vehicle	Public transport					

Section B: ESET Questionnaire - Job Satisfaction Assessment

Instructions: For each part below, you are asked about your satisfaction or unhappiness of your job or career.

Please circle the relevant number as stated below. Please give a response by which you.

1- Fully disagree, 2 – Partly disagree, 3 – Neither agree nor disagree, 4 – Partly agree, 5 – Fully agree

11. I am satisfied with possibility for communication with health staff including nursing staff of my place of Service.	1	2	3	4	5
12. I am satisfied with the cooperation in between me and health staff	1	2	3	4	5
13. I am satisfied with load of works to be done by me within my ward/ unit.	1	2	3	4	5
14. I am satisfied with courage received from health staff to provide more suitable care to patients	1	2	3	4	5
15. I am satisfied with attempts made by superior officers to upgrade my service condition	1	2	3	4	5
16. I am satisfied with my taking part in decision making within my ward/ unit	1	2	3	4	5
17. I am satisfied with the ratio of nursing/ patients in one duty shift	1	2	3	4	5
18. I am satisfied with the opportunity received to improve my career status	1	2	3	4	5
19. I am satisfied with confidence I have on other staff of the ward	1	2	3	4	5
20. I am satisfied with opportunities for training courses given to me by my superior officers	1	2	3	4	5
21. I am satisfied with number of nursing officers available for the service according to load of works to be done in one duty shift	1	2	3	4	5
22. I am satisfied with physical condition within my ward/ unit	1	2	3	4	5
23. I am satisfied with the daily routing of my ward/ unit	1	2	3	4	5
24. I am satisfied with appreciations received from my superior officers for my service	1	2	3	4	5
25. I am satisfied with appreciation of my service by patients	1	2	3	4	5
26. I am satisfied with appreciation of my works by patients and their family members	1	2	3	4	5

27. I am satisfied with my nursing role within the ward	1	2	3	4	5

28. I am satisfied with talents of other health professionals	1	2	3	4	5
29. I am satisfied with opportunities given to me by my unit to take part in training courses	1	2	3	4	5
30. I am satisfied with career competency of my colleagues	1	2	3	4	5
31. I am satisfied with opportunities to use novel knowledge with my ward/ unit	1	2	3	4	5
32. I am satisfied with sufficiency of tools available within my ward/ unit	1	2	3	4	5
33. I am satisfied with organizational structure of my place of work	1	2	3	4	5
34. I am satisfied with independence I have in giving patients a sufficient service based on my capabilities	1	2	3	4	5
35. I am satisfied with the protocols organized within my ward/ unit	1	2	3	4	5
36. I am satisfied with the quality of my unit/ ward and service provided by me	1	2	3	4	5
37. I am satisfied with the period of time for a promotion within my place of service	1	2	3	4	5
38. I am satisfied with works done in my unit/ ward	1	2	3	4	5
39. I am satisfied with respect received from my superior officers regarding my service	1	2	3	4	5
40. I am satisfied with the number of protocols available for functioning of my unit/ ward	1	2	3	4	5
41. I am satisfied with the salary received for my service	1	2	3	4	5
42. I am satisfied with my salary in accordance with my knowledge/ competency	1	2	3	4	5
43. I am satisfied with opportunities I have to share Information with my superior officers.	1	2	3	4	5
44. I am satisfied with ideas/ feelings of patients regarding my activities/ service	1	2	3	4	5
45. I am satisfied with encouragements by my superior officers to training sessions	1	2	3	4	5
46. I am satisfied with respect from patients regarding my taking actions	1	2	3	4	5
47. I am satisfied with respect from other health staff/health professionals regarding my taking actions	1	2	3	4	5

Section C: Work-related Factors

Instructions: Please answer all the questions.

48. Average number of leaves per month	
49. Average number of OT hours per month	
50. Average number of absentees per year	

Below are a series of statements concerning your thoughts/findings about your job.

Please circle the most appropriate response.

1- Fully disagree, 2 – Partly disagree, 3 – Neither agree nor disagree, 4 – Partly agree, 5 – Fully agree

51. I'm satisfied with nurse patient ratio	1	2	3	4	5
52. I'm satisfied with facilities at workplace	1	2	3	4	5
53. I'm satisfied with peer support	1	2	3	4	5
54. I have good job qualifications to give good care for patients	1	2	3	4	5

Section D: Socio-economic Factors

Instructions: Please answer all the questions.

55. Total income of the family				
56. Your monthly income				
57. Average time spending to come to work		Hours		Minutes
58. Average monthly expenditure for transport				
